# Exploring the risk factors of impaired fasting glucose in middle-aged population living in South Korean communities by using categorical boosting machine

**DOI:** 10.3389/fendo.2022.1013162

**Published:** 2022-09-29

**Authors:** Haewon Byeon

**Affiliations:** ^1^ Department of Digital Anti-aging Healthcare (BK21), Graduate School of Inje University, Gimhae, South Korea; ^2^ Department of Medical Big Data, College of AI Convergence, Inje University, Gimhae, South Korea

**Keywords:** impaired fasting glucose, risk factor, CatBoost, machine learning, middle-aged population

## Abstract

**Objective:**

This epidemiological study (1) identified factors associated with impaired fasting glucose using 3,019 subjects (≥30 years old and <60 years old) without diabetes mellitus from national survey data and (2) developed a nomogram that could predict groups vulnerable to impaired fasting glucose by using machine learning.

**Methods:**

This study analyzed 3,019 adults between 30 and 65 years old who completed blood tests, physical measurements, blood pressure measurements, and health surveys. Impaired fasting glucose, a dependent variable, was classified into normal blood glucose (glycated hemoglobin<5.7% and fasting blood glucose ≤ 100mg/dl) and impaired fasting glucose (glycated hemoglobin is 5.7-6.4% and fasting blood glucose is 100-125mg/dl). Explanatory variables included socio-demographic factors, health habit factors, anthropometric factors, dietary habit factors, and cardiovascular disease risk factors. This study developed a model for predicting impaired fasting glucose by using logistic nomogram and categorical boosting (CatBoost).

**Results:**

In this study, the top eight variables with a high impact on CatBoost model output were age, high cholesterol, WHtR, BMI, drinking more than one shot per month for the past year, marital status, hypertension, and smoking.

**Conclusion:**

It is necessary to improve lifestyle and continuously monitor subjects at the primary medical care level so that we can detect non-diabetics vulnerable to impaired fasting glucose living in the community at an early stage and manage their blood glucose.

## Introduction

The number of diabetic patients is increasing rapidly worldwide. The International Diabetes Federation predicted that if the current rate of increase would continue, the number of people with diabetes will increase from 425 million at present to approximately 700 million in 2045 (1). It is also estimated that diabetes prevalence will rapidly increase in South Korea as well and the number of people with diabetes will exceed 6 million around 2050 (2). As the prevalence of diabetes has skyrocketed explosively in Asia as well as North and South America in recent years, it has become an important issue in health science.

Diabetes mellitus is a disease that maintains a high blood glucose level for a long period (3–5). It is known to cause various complications such as diabetic retinopathy and kidney disease because it leads to disorders in microvessels such as the retina or kidneys (3–5). It is also reported that 1 million people die each year worldwide from diabetes mellitus and its complications (3, 6). Therefore, the prevention and continuous management of diabetes mellitus have emerged as important health issues after middle age (3, 6). Diabetes mellitus can be divided into type 1 diabetes mellitus and type 2 diabetes mellitus (7, 8). Type 1 diabetes mellitus occurs because the beta cells of the pancreas are destroyed by the immune system and are unable to secrete insulin (7, 8). Type 2 diabetes mellitus is caused by relatively increased insulin resistance due to various causal factors such as health habits, even though insulin-secreting function remains partially (7, 8). In South Korea, 97% of diabetic patients are diagnosed with type 2 diabetes mellitus. The known key causal factors of type 2 diabetes mellitus are aging, obesity, dietary habits (e.g., excessive intake of simple sugar, high calorie, and high-fat food), and living habits (e.g., insufficient exercise and curtailed sleep) in addition to family medical history (9–14).

Although the prevalence of diabetes mellitus has steadily increased over the past 20 years, it is difficult to detect diabetes mellitus early because there are almost no subjective symptoms unless the blood glucose level is too high (15). Ramachandran (2014) (16) reported that many diabetic patients who were diagnosed with diabetes mellitus at a health medical examination already had vascular complications. In particular, when fasting blood glucose is between 100 and 125mg/dL, which is an intermediate level between normal and diabetes, or when glycated hemoglobin is between 5.7 and 6.4%, it is diagnosed with impaired fasting glucose (17). Since people satisfying these criteria are more likely to develop diabetes in the future, it is defined as prediabetes (17).

Diabetes Fact Sheets in Korea (2021) (15) reported that as of 2021, one in seven (13.8%) adults (≥30 years old) suffered from diabetes mellitus and one in four (26.9%) adults were prediabetes. However, 35% of patients who were diagnosed with diabetes mellitus between 2016 and 2018 did not have a subjective symptom (15). Therefore, to prevent diabetes, it requires to detect impaired fasting glucose, the pre-diabetes stage, as soon as possible, regulate diet (e.g., proper eating habits), improve lifestyle habits (e.g., exercise and weight control), and monitor the progress to diabetes by conducting a blood glucose test regularly (18). This epidemiological study (1) identified factors associated with impaired fasting glucose using 3,019 subjects (≥30 years old and <60 years old) without diabetes mellitus from national survey data and (2) developed a nomogram that could predict groups vulnerable to impaired fasting glucose by using machine learning.

## Methods and materials

### Subjects

This study used secondary data based on the raw data of the 2020 Korea National Health and Nutrition Examination Survey (KNHANES), a national statistic (No. 117002), supervised by the Ministry of Health and Welfare and the Korea Centers for Disease Control and Prevention. The KNHANES was approved by the Institutional Review Board of the Korea Centers for Disease Control and Prevention (No. 2018-01-03-2C-A). Moreover, de-identification measures were applied to some variables such as health insurance types for protecting personal information. The population of the KNHANES was the people living in South Korea. It chose study subjects (samples) from the Population and Housing Census (complete enumeration survey) data by using the stratified cluster sampling method and the systematic sampling method. The 2020 KNHANES targeted 9,949 people in 192 sampling districts across the country. However, the health and examination survey completed only 7,359 people (participation rate=74.0%) in 180 sampling districts due to the suspension of the investigation caused by the COVID-19 pandemic. The health survey of the KNHANES was conducted by face-to-face interviews and self-reporting after a surveyor visited the target household. The examination consists of blood pressure measurement, physical measurement, and blood test. During the survey period, medical workers (e.g., doctors and nurses) conducted a 1:1 examination and health survey by visiting the survey area using a mobile examination vehicle. This study excluded pregnant women at the time of the survey and those who were already diagnosed with diabetes. This study analyzed 3,019 adults between 30 and 65 years old who completed blood tests, physical measurements, blood pressure measurements, and health surveys.

### Measurement and definition of variables

Impaired fasting glucose, a dependent variable, was classified into normal blood glucose (glycated hemoglobin<5.7% and fasting blood glucose ≤ 100mg/dl) and impaired fasting glucose (glycated hemoglobin is 5.7-6.4% and fasting blood glucose is 100-125mg/dl) based on the Clinical Practice Guidelines of the Korean Diabetes Association (2021) (19) according to the diagnosis of medical personnel. For the sample container, NaF, SST, and EDTA tubes were used for a blood glucose test, a general blood chemistry test, and a hematology test, respectively. They were processed in accordance with the specimen storage and separation regulations. Fasting blood glucose, HbA1c, insulin, triglycerides, and total cholesterol were measured with the separated plasma and serum. This study measured fasting blood glucose with Pureauto S GLU (Hexokinase UV method), triglyceride with Pureauto S TG-N (enzyme method), and total cholesterol with Pureauto SCHO-N (Daiichi Pure Chemicals Corporation, Tokyo, Japan) by using Hitachi 7600-210 (Hitachi high-technologies Co., Tokyo, Japan), an automated analyzer for blood tests.

Explanatory variables included socio-demographic factors, health habit factors, anthropometric factors, dietary habit factors, and cardiovascular disease risk factors, referring to previous studies (9–14). Socio-demographic factors included gender (male or female), marital status (living with a spouse, separated/divorced/bereaved, or single), age (30-39, 40-49, or 50-64), living area (urban or rural), and monthly mean household income (<2 million KRW, 2-4 million KRW, or >4 million KRW). Health habit factors included drinking more than one shot per month for the past year (yes or no), smoking (non-smoker, former smoker, or smoker), subjective stress level (almost none, moderate, or high), mean length of moderate level physical activities during leisure activities per day (activities that make a person slightly short of breath or the heart slightly faster, such as jogging or strength training: none, 10 minutes≤ and <1 hour, or 1 hour≤), mean daily sitting time (≤4 hours, 5 hours ≤ and ≤ 7 hours, or 8 hours≤), number of days of walking for more than 10 minutes per day in the past week (none, 1-2 days, 3-4 days, 5-6 days, or every day), and weekly mean sleeping hours per day (≤5 hours, 6-7 hours, or 8 hours≤). Anthropometric factors included body mass index (BMI: underweight (<18.5kg/m2), normal weight (18.5-23kg/m2), pre-obesity class (23-25kg/m2), class 1 obesity (25-30kg/m2), or class 2 obesity or higher (>30kg/m2)) and waist-to-height ratio (WHtR: <0.5 or 0.5≤ (Zeng et al. (2014) (20)). Dietary habit factors included the mean number of days of having breakfast per week for the past year (5-7 days per week, 3-4 days per week, 1-2 days per week, or rarely) and the mean frequency of eating out including delivery for the past year (<1 per day or 1 per day≤). For cardiovascular disease risk factors, this study referred to systolic blood pressure and total cholesterol, the criteria announced by the American Heart Association (21) used to calculate cardiovascular disease risk, and triglyceride and diastolic blood pressure, which were factors related to the risk of cardiovascular disease in Kim & Ryu (2018) (22). The cardiovascular disease risk factors of this study included high cholesterol prevalence (total cholesterol≥240mg/dL: yes or no), high triglyceride prevalence (triglyceride content≥200mg/dL: yes or no), hypertension prevalence ((1) normal blood pressure: systolic blood pressure < 120mmHg and diastolic blood pressure < 80mmHg, (2) pre-hypertension: systolic blood pressure is between 120 and 140mmHg and diastolic blood pressure is 80-90mmHg, and (3) hypertension: systolic blood pressure > 120mmHg and diastolic blood pressure > 80mmHg). Blood pressure was measured on the right upper arm using a mercury sphygmomanometer (Wall Unit 33, Baumanometer, America) after the subject rested for five minutes under the supervision of the nurse in charge (23).

### Variable selection

CatBoost (category boosting) is an algorithm developed by Yandex Technologies and is known to be useful for processing categorical variables (24). Previously developed gradient boosting techniques such as XGBoost and LightGBM have two shortfalls. First, as the learning of boosting progresses, the distribution of data is changed. As a result, prediction shift that leads to overfitting problems or inaccurate predictions occurs. Second, it takes a lot of time to process categorical variables. For example, when XGBoost and LightGBM newly create binary variables, the amount of statistics increases, and, as a result, computation time and memory consumption increase. CatBoost constructs a model using ordered boosting to overcome these limitations. Existing boosting models (e.g., XGBoost and LightGBM) build models by calculating residuals for all training data. However, CatBoost calculates residuals by using only a portion of the training data, as shown in Equation (1), and builds a model based on them. Afterward, the residual of the data reuses the value predicted by this model.


(1) $$input\;:\;{\left\{\left(X_{k},\;Y_{k}\right)\right\}}_{k=1}^{n}\;\text{ordered according to σ},\text{ the number of trees }I;$$

σ←random permutation of [1,n];

$$M_{i}\leftarrow 0\text{ for }i=1,\;\cdots ,\;n;$$

for t←1 to I do

for i←1 to n do

$$r_{i}\leftarrow y_{i}-M_{\sigma \left(i\right)-1}\left(X_{i}\right);$$

for i←1 to n do

$$\Delta M\leftarrow LearnModel\left[\left(X_{i},r_{j}\right)\;:\sigma \left(j\right)\leq i\right]$$

$$M_{i}\leftarrow M_{i}+\Delta M$$

$$\text{return }M_{n}$$

CatBoost is easier to use than other gradient boosting algorithms because it optimizes hyperparameters by using an internal algorithm without a special hyperparameter optimization process, its advantage. For CatBoost, this study set the number of trees to 100, the Lambda of regularization to 3, the learning rate to 0.300, and the limit depth of individual trees to 6. The CatBoost algorithm calculates feature importance by using a mean decrease in impurity to select important variables for predicting impaired fasting glucose. This study developed a nomogram for predicting groups vulnerable to impaired fasting glucose by selecting the eight variables with the highest feature importance for interpreting risk probabilities efficiently.

### Development a nomogram for predicting impaired fasting glucose

This study developed a model for predicting impaired fasting glucose by using logistic regression analysis to identify the independent relevance of variables related to impaired fasting glucose in people without diabetes residing in the local community by entering the top variables with high feature importance found in CatBoost. The regression model presented a 95% confidence interval (CI) and adjusted odds ratio (aOR), which adjusted for all confounding factors. A nomogram was developed based on the developed predictive model (final model) for impaired fasting glucose so that medical personnel could easily interpret the prediction result (prediction probability). The nomogram was composed of four components: a point line, a risk factor line, a probability line, and a total point line. Please refer to Byeon (2022) (25) for a detailed description of the function and composition of the nomogram. The predictive performance of the nomogram for predicting impaired fasting glucose in non-diabetics was evaluated using F1-score, the area under the curve (AUC), precision, recall, general accuracy, and calibration plots. The final model was validated using 10-fold cross-validation. All analyses were performed using Python version 3.9.12 (https://www.python.org).

## Results

### General characteristics of subjects according to impaired fasting glucose

**Table 1** presents the results of the chi-square test, which analyzed the differences in general characteristics between groups according to the prevalence of impaired fasting glucose. Among 3,019 subjects, 879 subjects (29.1%) had impaired fasting glucose. The results of the chi-square test showed that the two groups were significantly different in marital status, age, mean monthly household income, drinking experience for the past year, subjective stress, the mean number of days of walking per week, weekly mean sleeping hours per day, BMI, WHtR, mean number of days of having breakfast per week for the past year, mean frequency of eating out including delivery for the past year, high cholesterol, high triglycerides, and hypertension (p<0.05).

**Table 1:** General characteristics of subjects according to the prevalence of impaired fasting glucose (n, %).

Variables	Impaired fasting glucose		p
	No (n = 2,140)	Yes (n = 879)
Gender			0.149
Male	898 (69.5)	394 (30.5)	
Female	1,242 (71.9)	485 (28.1)	
Marital Status			<0.001
Living with a spouse	1,614 (69.2)	719 (30.8)	
Separated/divorced/bereaved	190 (65.7)	99 (34.3)	
Single	333 (84.5)	61 (15.5)	
Age			<0.001
30-39	647 (87.9)	89 (12.1)	
40-49	706 (78.8)	190 (21.2)	
50-64	787 (56.7)	600(43.3)	
Living area			0.758
Urban	1,763 (71.0)	720 (29.0)	
Rural	377 (70.3)	159 (29.7)	
Monthly mean household income			0.029
<2 million KRW	267 (66.4)	135 (33.6)	
2-4 million KRW	506 (69.2)	225 (30.8)	
>4 million KRW	1,364 (72.4)	519 (27.6)	
Drinking experience for the past year			<0.001
No	833 (66.2)	425 (33.8)	
Yes	1,298 (74.3)	449 (25.7)	
Smoking			0.392
Non-smoker	1,268 (71.6)	504 (28.4)	
Former smoker	486 (71.2)	197 (28.8)	
Smoker	377 (68.5)	173 (31.5)	
Subjective stress			0.008
Almost none	206 (64.6)	113 (35.4)	
Moderate	1,239 (70.6)	515 (29.4)	
High	686 (73.7)	245 (26.3)	
Mean length of moderate level physical activities per day			0.157
None	1,419 (69.7)	618 (30.3)	
<1 hour	379 (73.9)	134 (26.1)	
1 hour≤	228 (71.7)	90 (28.3)	
Mean daily sitting time			0.056
≤4 hours	334 (69.6)	146 (30.4)	
5-7 hours	518 (67.7)	247 (32.3)	
8 hours≤	1,171 (72.4)	447 (27.6)	
Mean number of days of walking per week			0.028
None	360 (68.3)	167 (31.7)	
1-2 days	408 (75.1)	135 (24.9)	
3-4 days	392 (68.3)	182 (31.7)	
5-6 days	363 (73.3)	132 (26.7)	
7 days (every day)	503 (69.0)	226 (31.0)	
Weekly mean sleeping hours per day			0.006
≤5 hours	310 (64.9)	168 (35.1)	
6-7 hours	1,224 (71.6)	485 (28.4)	
8 hours≤	604 (72.8)	226 (27.2)	
BMI			<0.001
Underweight	91 (85.8)	15 (14.2)	
Normal weight	866 (79.2)	228 (20.8)	
Pre-obesity class	489 (70.6)	203 (29.4)	
Class 1 obesity	588 (62.9)	347 (37.1)	
Class 2 obesity or higher	89 (52.4)	81 (47.6)	
WHtR			<0.001
<0.5	1,103 (81.6)	249 (18.4)	
0.5≤	1,018 (62.0)	625 (38.0)	
Mean number of days of having breakfast per week for the past year			<0.001
5-7 days per week	825 (67.5)	398 (32.5)	
3-4 days per week	176 (71.8)	69 (28.2)	
1-2 days per week	258 (77.9)	73 (22.1)	
Rarely	402 (77.3)	118 (22.7)	
Mean frequency of eating out including delivery for the past year			0.009
1 per day≤	446 (75.9)	142 (24.1)	
<1 per day	1,215 (70.2)	516 (29.8)	
High cholesterol			<0.001
No	1,748 (76.1)	548 (23.9)	
Yes	345 (52.8)	308 (47.2)	
High triglyceride			<0.001
No	1,486 (72.4)	567 (27.6)	
Yes	232 (62.0)	142 (38.0)	
Hypertension			<0.001
Normal	1,120 (79.6)	287 (20.4)	
Pre-hypertension	570 (67.9)	269 (32.1)	
Hypertension	416 (57.5)	307 (42.5)	

### Predictors for impaired fasting glucose in non-diabetes living in local communities in South Korea

**Figure 1:**
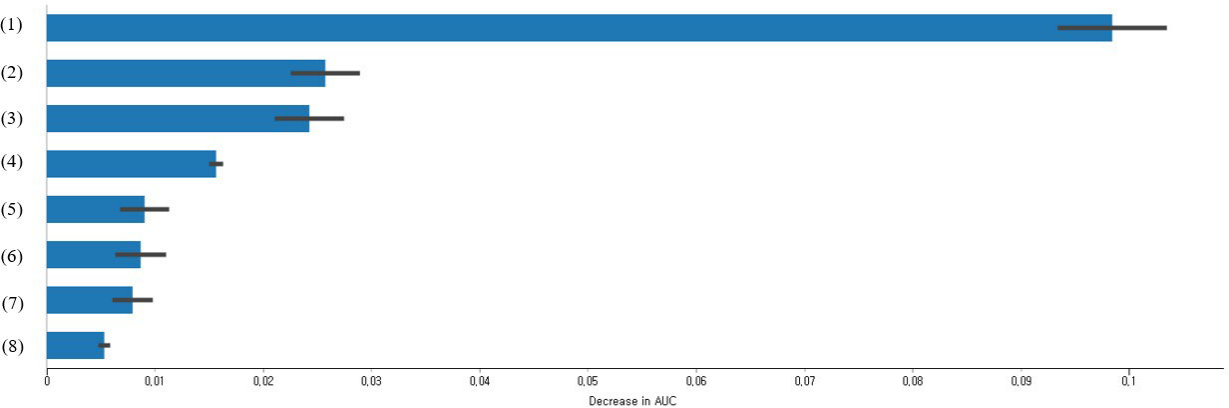
Feature importance of impaired fasting glucose predictors in non-diabetics based on CatBoost (only the top eight variables are presented); (1) Age (1 = 30-39 years old, 2 = 40-49 years old, or 3 = 50-64 years old); (2) High cholesterol (0 = no or 1 = yes); (3) WHtR (1 = <0.5 or 2 = 0.5≤); (4) BMI (1 = underweight, 2 = normal weight, 3 = pre-obesity class, 4 = class 1 obesity, or 5 = class 2 obesity or higher); (5) Drinking more than one shot per month for the past year (0 = no or 1 = yes); (6) Marital status (1 = living with a spouse, 2 = separated/divorced/bereaved, or 3 = single); (7) Hypertension (1 = normal, 2 = pre-hypertension, or 3 = hypertension); and (8) Smoking (1 = non-smoker, 2 = former smoker, or 3 = smoker).

**Figure 1** shows the calculated feature importance of factors related to impaired fasting glucose in people without diabetes using CatBoost. The results of this study revealed that age, high cholesterol, WHtR, BMI, drinking more than one shot per month for the past year, marital status, hypertension, and smoking were the top eight variables based on feature importance.

**Table 2** shows the results of the logistic regression analysis for predicting impaired fasting glucose in South Korean non-diabetics using the top eight variables with high importance obtained from CatBoost. The analysis results of the adjusted model confirmed that separated/divorced/bereaved from their spouse (AOR=1.79, 95% CI: 1.26, 2.55), age (40-49 years old:AOR=1.59, 50-64 years old:AOR=4.09), no drinking experience for the past year (AOR=1.47, 95% CI: 1.21, 1.77), smoker (AOR=1.36, 95% CI: 1.06, 1.74), class 2 obesity or higher base on BMI (AOR=3.80, 95% CI: 1.80, 8.01), WHtR≥0.5 (AOR=1.37, 95% CI: 1.04, 1.81), high cholesterol (AOR=2.03, 95% CI: 1.66, 2.49) hypertension (pre-hypertension:AOR=1.34, hypertension:AOR=1.31) were the independent influencing factors of impaired fasting glucose (p<0.05).

**Table 2:** Predictors for impaired fasting glucose in non-diabetics living in local communities in South Korea: aOR and 95% CI.

Variables	aOR	95% CI	p
Marital Status			
Living with a spouse (ref)	1	1	
Separated/divorced/bereaved	1.79	1.26, 2.55	0.001
Single	1.47	0.94, 2.30	0.084
Age			
30-39(ref)	1	1	
40-49	1.59	1.17, 2.16	0.003
50-64	4.09	3.04, 5.49	<0.001
Drinking experience for the past year			
No	1.47	1.21, 1.77	<0.001
Yes (ref)	1	1	
Smoking			
Non-smoker (ref)	1	1	
Former smoker	0.97	0.77, 1.22	0.818
Smoker	1.36	1.06, 1.74	0.015
BMI			
Underweight (ref)	1	1	
Normal weight	1.10	0.59. 2.06	0.743
Pre-obesity class	1.22	0.63, 2.35	0.538
Class 1 obesity	1.69	0.86, 3.30	0.123
Class 2 obesity or higher	3.80	1.80, 8.01	<0.001
WHtR			
<0.5 (ref)	1	1	
0.5≤	1.37	1.04, 1.81	0.023
High cholesterol			
No (ref)	1	1	
Yes	2.03	1.66, 2.49	<0.001
Hypertension			
Normal (ref)	1	1	
Pre-hypertension	1.34	1.07, 1.67	0.008
Hypertension	1.31	1.03, 1.66	0.024

### Development and validation of the nomogram for predicting groups vulnerable to impaired fasting glucose in South Korean non-diabetics

**Figure 2:**
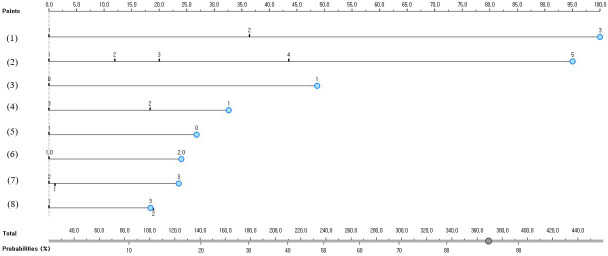
A nomogram for predicting impaired fasting glucose predictors in South Korean non-diabetics; (1) Age (1 = 30-39 years old, 2 = 40-49 years old, or 3 = 50-64 years old); (2) BMI (1 = underweight, 2 = normal weight, 3 = pre-obesity class, 4 = class 1 obesity, or 5 = class 2 obesity or higher); (3) High cholesterol (0 = no or 1 = yes); (4) Marital status (1 = living with a spouse, 2 = separated/divorced/bereaved, or 3 = single); (5) Drinking more than one shot per month for the past year (0 = no or 1 = yes); (6) WHtR (1 = <0.5 or 2 = 0.5≤); (7) Smoking (1 = non-smoker, 2 = former smoker, or 3 = smoker); and (8) Hypertension (1 = normal, 2 = pre-hypertension, or 3 = hypertension).

**Figure 2** presents the nomogram for predicting impaired fasting glucose in South Korean non-diabetics. The nomogram predicted that those who lived with a spouse, were class 2 obesity or higher based on BMI (>30kg/m2), had hypertension and high cholesterol, did not drink in the past one year, were smoking, and were between 50 and 64 years old had an 87% chance to have impaired fasting glucose (a high-risk group).

**Figure 3:**
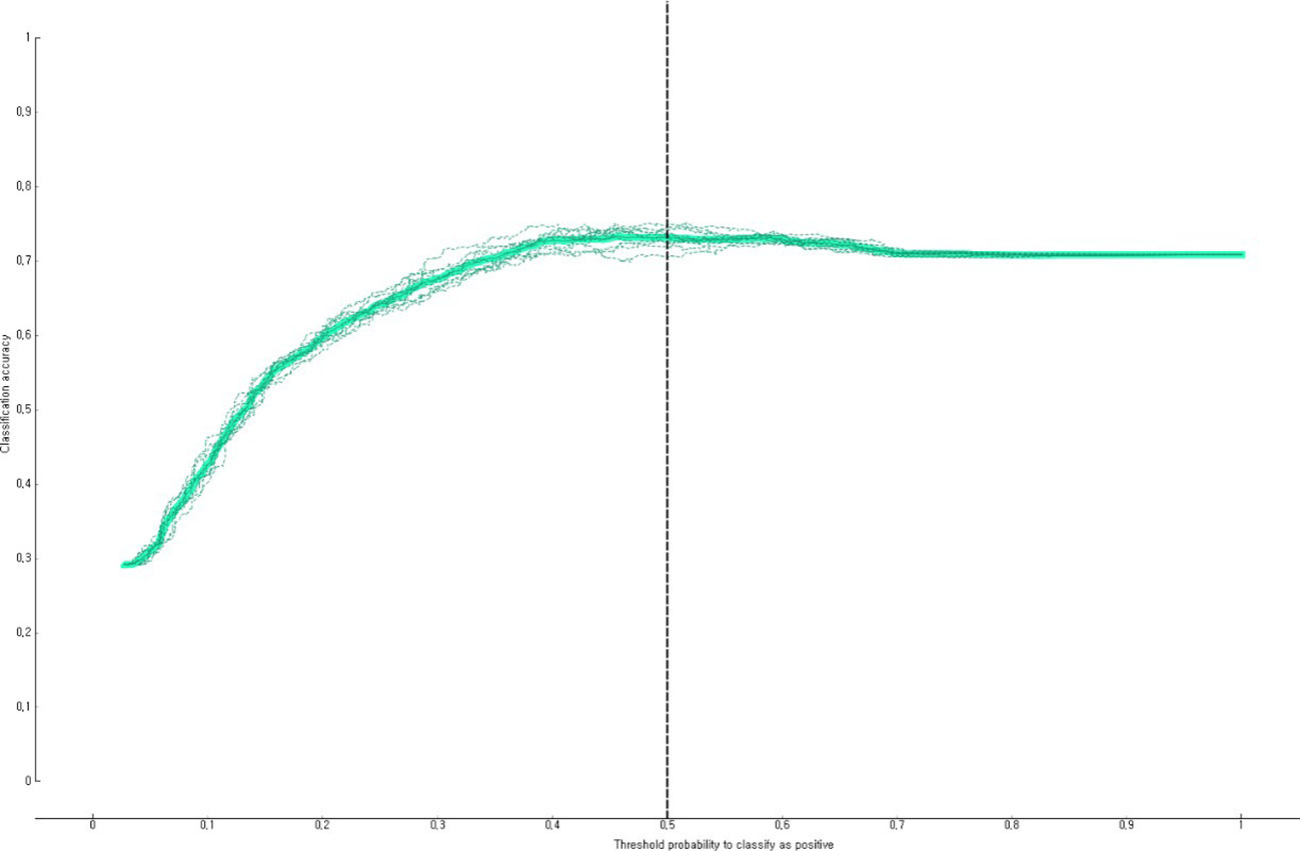
Accuracy of the nomogram for predicting impaired fasting glucose in South Korean non-diabetics.

**Figure 4:**
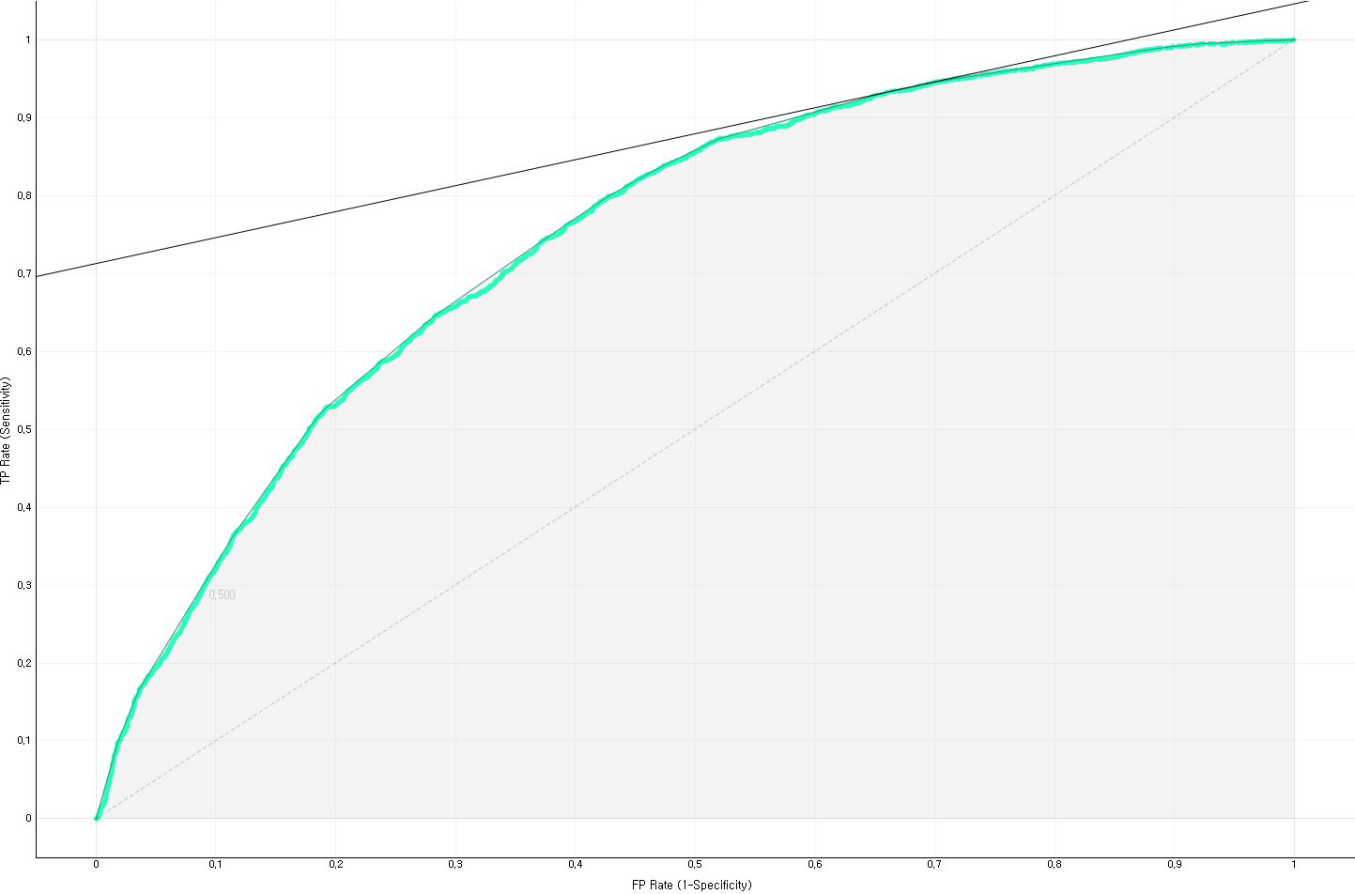
AUC of the nomogram for predicting impaired fasting glucose in South Korean non-diabetics.

**Figure 5:**
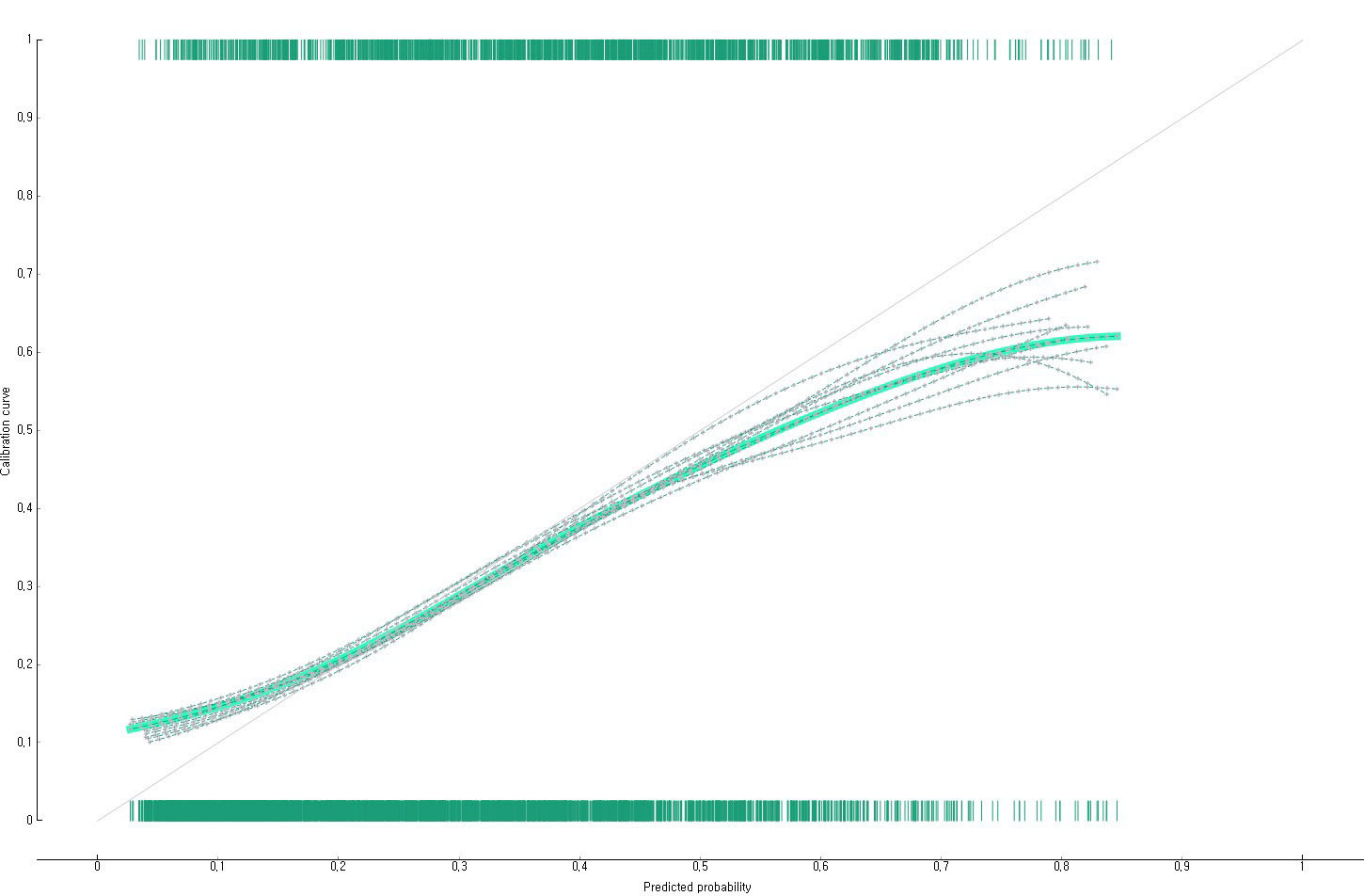
Calibration plot of the nomogram for predicting impaired fasting glucose in South Korean non-diabetics.

The predictive performance of the developed impaired fasting glucose prediction nomogram was validated by using general accuracy (**Figure 3**), AUC (**Figure 4**), precision, recall, F1-score (2(precision＊recall)/precision+recall), and calibration plot (**Figure 5**). A calibration plot (**Figure 5**) and the chi-square test showed that the predicted and observed probabilities of the impaired fasting glucose group and the normal blood glucose group were not significantly different (p<0.05). The results of 10-fold cross validation revealed that the general accuracy, AUC, precision, recall, and F1-score of the nomogram for predicting impaired fasting glucose in non-diabetics were 0.73, 0.75, 0.71, 0.73, and F1-score, respectively.

## Discussion

This study identified factors related to impaired fasting glucose in non-diabetics according to the diagnostic criteria for impaired fasting glucose suggested by the Korean Diabetes Association by using epidemiological data representing the South Korean population. It was confirmed that age, high cholesterol, WHtR, BMI, drinking more than one shot per month for the past year, marital status, hypertension, and smoking were factors independently related to impaired fasting glucose. Many previous studies (26–31) also reported that age, high cholesterol, hypertension, abdominal obesity, smoking experience (past and current smoking), and drinking were risk factors for pre-diabetes, which agreed with the results of this study. Socio-demographic factors such as age and marital status were identified as major risk factors for impaired fasting glucose in previous studies (32–34). Kwon & Na (2017) (32) reported that the risk of pre-diabetes also significantly increased as the age of subjects increased. Moreover, Cornelis et al. (2014) (34) revealed that men living with their spouses had a 16% higher risk of type 2 diabetes than men who did not live with a spouse. Socio-demographic factors such as marital status could increase the risk of diabetes by ultimately mediating non-lifestyle habits or obesity due to their impacts on dietary habits and other factors.

The results of this study showed that the risk of impaired fasting glucose increased when body fat distribution scales (e.g., WHtR and BMI) were higher, which were similar to the results of previous studies (35–38). Previous studies (35, 37, 39, 40) reported that obesity was a major cause of pre-diabetes and diabetes and obese adults had a high risk of pre-diabetes (35, 39, 40). Halpern & Mancini (2005) (41) revealed that 80% of patients with type 2 diabetes were obese.

In particular, it is noteworthy that many previous studies reported WHtR, a criterion for judging abdominal obesity, as a major factor influencing fasting blood glucose, which agreed with the results of this study (38, 42). Mi et al. (2013) (38) compared the odds ratio of type 2 diabetes with BMI, waist circumferences, WHtR, visceral fat index, and body fat index using 8,121 adults living in the local community and confirmed WHtR as the most effective screening index for type 2 diabetes. It is believed that it is because the risk of hyperinsulinemia increases when abdominal obesity is higher (43). Obesity excessively accumulates body fat in the abdomen, which increases the activity of hormone-sensitive fat enzymes in the adipose tissue and lipolysis (44). As a result, the concentration of free fatty acids increases (44). The increased free fatty acid lowers muscle glycogen synthesis by inhibiting the uptake and utilization of glucose in the muscle and liver (44). Furthermore, it worsens insulin resistance by affecting the insulin receptor in the muscle and liver (44). Consequently, adults with abdominal obesity are more likely to progress to impaired fasting glucose.

This study confirmed that hypertension and high cholesterol were also major risk factors for impaired fasting glucose. Emdin et al. (2015) (45) evaluated the relationship between blood pressure and the risk of type 2 diabetes using meta-analysis and reported that blood pressure and blood glucose were closely related ((1) when systolic blood pressure increased by 20mmHg increased, the risk of type 2 diabetes increased by 58% and (2) when diastolic blood pressure increased by 10mmHg, the risk of type 2 diabetes increased by 52%). In addition, Meikle et al. (2013) (46) also confirmed a significant positive correlation between total cholesterol concentration and type 2 diabetes. However, it is still limited to predicting impaired fasting glucose early while reflecting the actual characteristics of the local community population, which have diverse risk factors at the same time because there most of the previous studies that discovered the risk factors of impaired fasting glucose used regression analysis. To predict groups vulnerable to impaired fasting glucose early, it is necessary to conduct studies for identifying multiple risk factors for impaired fasting glucose based on real-world data.

Another finding of this study’s nomogram was that it predicted that those who lived with a spouse, were class 2 obesity or higher based on BMI (>30kg/m2), had hypertension and high cholesterol, did not drink in the past one year, were smoking, and were between 50 and 64 years old had an 87% chance to have impaired fasting glucose, which was very high. In South Korea, adults with impaired fasting glucose also receive a blood test (fasting glucose test) once a year using the National Health Screening Program, just like those with normal blood sugar. However, there is a limit to sensitively identifying the group vulnerable to diabetes just by using one fasting blood glucose test per year in the National Health Screening Program. To more sensitively identify groups vulnerable to diabetes through the National Health Screening Program in the future, it is needed to perform additional screening tests such as the oral glucose tolerance test or the glycated hemoglobin test for the group vulnerable to impaired fasting glucose found in this study. It is also necessary to improve the system so that groups vulnerable to impaired fasting glucose can receive a glycated hemoglobin test every 3 months through the National Health Screening Program. Since there are only a few previous studies that analyzed multiple health risk factors for impaired fasting glucose, additional epidemiological studies are required to analyze multiple health risk factors for impaired fasting glucose while considering various factors. It is also necessary to actively publicize and educate the risk of high blood glucose and measures to prevent diabetes for the group vulnerable to impaired fasting glucose.

This study has several limitations. First, since this epidemiological study analyzed cross-sectional data, it could not reveal a clear causal relationship. Additional prospective cohort studies are required to understand the causal relationship between the group vulnerable to impaired fasting glucose and impaired fasting glucose. Second, there may be potential factors of impaired fasting glucose not included in this study. The source data of this study did not investigate the nutrient intake ratio according to the frequency of food intake and family medical history. Future studies are needed to develop models to predict groups vulnerable to impaired fasting glucose while considering various potential variables highly related to fasting blood glucose, such as genetic data, nutrient intake ratio, and family medical history.

## Conclusions

This epidemiological study confirmed that age, high cholesterol, WHtR, BMI, drinking more than one shot per month for the past year, marital status, hypertension, and smoking were independently related to the impaired fasting glucose of non-diabetics. It is necessary to improve lifestyle and continuously monitor subjects at the primary medical care level so that we can detect non-diabetics vulnerable to impaired fasting glucose living in the community at an early stage and manage their blood glucose. Furthermore, it is needed to prepare a system that can actively implement additional screening tests such as an oral glucose tolerance test and a glycated hemoglobin test for groups vulnerable to impaired fasting glucose for preventing diabetes and detecting diabetes early at the community level. Additional longitudinal studies are required to confirm the causality between impaired fasting glucose and high-risk factors related to impaired fasting glucose identified in this study.

## Data availability statement

The raw data supporting the conclusions of this article will be made available by the authors, without undue reservation.

## Ethics statement

The study was conducted according to the guidelines of the Declaration of Helsinki and approved by the Institutional Review Board (or Ethics Committee) of Korea Disease Control and Prevention Agency (protocol code 2018-01-03-2C-A and date: 2020.05.01). The patients/participants provided their written informed consent to participate in this study.

## Author contributions

The author confirms being the sole contributor of this work and has approved it for publication.

## Funding

This research was supported by Basic Science Research Program through the National Research Foundation of Korea (NRF) funded by the Ministry of Education (NRF- 2018R1D1A1B07041091, 2021S1A5A8062526) and 2022 Development of Open-Lab based on 4P in the Southeast Zone.

## Conflict of interest

The author declares that the research was conducted in the absence of any commercial or financial relationships that could be construed as a potential conflict of interest.

## Publisher’s note

All claims expressed in this article are solely those of the authors and do not necessarily represent those of their affiliated organizations, or those of the publisher, the editors and the reviewers. Any product that may be evaluated in this article, or claim that may be made by its manufacturer, is not guaranteed or endorsed by the publisher.
