# Supplementary material for: Exploring the risk factors of impaired fasting glucose in middle-aged population living in South Korean communities by using categorical boosting machine
Source: Front Endocrinol (Lausanne). 2022 Sep 29;13:1013162. doi: 10.3389/fendo.2022.1013162 (PMC9556903; doi:10.3389/fendo.2022.1013162)
Supplement: Supplementary file 1 [file DataSheet_1.docx]

Supplementary Material

# Supplementary Tables

**Table 1**. General characteristics of subjects according to the prevalence of impaired fasting glucose (n, %)

| **Variables** | **Impaired fasting glucose** | | **p** |
| --- | --- | --- | --- |
|  | **No (n=2,140)** | **Yes (n=879)** |  |
| Gender |  |  | 0.149 |
| Male | 898 (69.5) | 394 (30.5) |  |
| Female | 1,242 (71.9) | 485 (28.1) |  |
| Marital Status |  |  | <0.001 |
| Living with a spouse | 1,614 (69.2) | 719 (30.8) |  |
| Separated/divorced/bereaved | 190 (65.7) | 99 (34.3) |  |
| Single | 333 (84.5) | 61 (15.5) |  |
| Age |  |  | <0.001 |
| 30-39 | 647 (87.9) | 89 (12.1) |  |
| 40-49 | 706 (78.8) | 190 (21.2) |  |
| 50-64 | 787 (56.7) | 600(43.3) |  |
| Living area |  |  | 0.758 |
| Urban | 1,763 (71.0) | 720 (29.0) |  |
| Rural | 377 (70.3) | 159 (29.7) |  |
| Monthly mean household income |  |  | 0.029 |
| <2 million KRW | 267 (66.4) | 135 (33.6) |  |
| 2-4 million KRW | 506 (69.2) | 225 (30.8) |  |
| >4 million KRW | 1,364 (72.4) | 519 (27.6) |  |
| Drinking experience for the past year |  |  | <0.001 |
| No | 833 (66.2) | 425 (33.8) |  |
| Yes | 1,298 (74.3) | 449 (25.7) |  |
| Smoking |  |  | 0.392 |
| Non-smoker | 1,268 (71.6) | 504 (28.4) |  |
| Former smoker | 486 (71.2) | 197 (28.8) |  |
| Smoker | 377 (68.5) | 173 (31.5) |  |
| Subjective stress |  |  | 0.008 |
| Almost none | 206 (64.6) | 113 (35.4) |  |
| Moderate | 1,239 (70.6) | 515 (29.4) |  |
| High | 686 (73.7) | 245 (26.3) |  |
| Mean length of moderate level physical activities per day |  |  | 0.157 |
| None | 1,419 (69.7) | 618 (30.3) |  |
| <1 hour | 379 (73.9) | 134 (26.1) |  |
| 1 hour≤ | 228 (71.7) | 90 (28.3) |  |
| Mean daily sitting time |  |  | 0.056 |
| ≤4 hours | 334 (69.6) | 146 (30.4) |  |
| 5-7 hours | 518 (67.7) | 247 (32.3) |  |
| 8 hours≤ | 1,171 (72.4) | 447 (27.6) |  |
| Mean number of days of walking per week |  |  | 0.028 |
| None | 360 (68.3) | 167 (31.7) |  |
| 1-2 days | 408 (75.1) | 135 (24.9) |  |
| 3-4 days | 392 (68.3) | 182 (31.7) |  |
| 5-6 days | 363 (73.3) | 132 (26.7) |  |
| 7 days (every day) | 503 (69.0) | 226 (31.0) |  |
| Weekly mean sleeping hours per day |  |  | 0.006 |
| ≤5 hours | 310 (64.9) | 168 (35.1) |  |
| 6-7 hours | 1,224 (71.6) | 485 (28.4) |  |
| 8 hours≤ | 604 (72.8) | 226 (27.2) |  |
| BMI |  |  | <0.001 |
| Underweight | 91 (85.8) | 15 (14.2) |  |
| Normal weight | 866 (79.2) | 228 (20.8) |  |
| Pre-obesity class | 489 (70.6) | 203 (29.4) |  |
| Class 1 obesity | 588 (62.9) | 347 (37.1) |  |
| Class 2 obesity or higher | 89 (52.4) | 81 (47.6) |  |
| WHtR |  |  | <0.001 |
| <0.5 | 1,103 (81.6) | 249 (18.4) |  |
| 0.5≤ | 1,018 (62.0) | 625 (38.0) |  |
| Mean number of days of having breakfast per week for the past year |  |  | <0.001 |
| 5-7 days per week | 825 (67.5) | 398 (32.5) |  |
| 3-4 days per week | 176 (71.8) | 69 (28.2) |  |
| 1-2 days per week | 258 (77.9) | 73 (22.1) |  |
| Rarely | 402 (77.3) | 118 (22.7) |  |
| Mean frequency of eating out including delivery for the past year |  |  | 0.009 |
| 1 per day≤ | 446 (75.9) | 142 (24.1) |  |
| <1 per day | 1,215 (70.2) | 516 (29.8) |  |
| High cholesterol |  |  | <0.001 |
| No | 1,748 (76.1) | 548 (23.9) |  |
| Yes | 345 (52.8) | 308 (47.2) |  |
| High triglyceride |  |  | <0.001 |
| No | 1,486 (72.4) | 567 (27.6) |  |
| Yes | 232 (62.0) | 142 (38.0) |  |
| Hypertension |  |  | <0.001 |
| Normal | 1,120 (79.6) | 287 (20.4) |  |
| Pre-hypertension | 570 (67.9) | 269 (32.1) |  |
| Hypertension | 416 (57.5) | 307 (42.5) |  |

**Table 2.** Predictors for impaired fasting glucose in non-diabetics living in local communities in South Korea: aOR and 95% CI

| **Variables** | **aOR** | **95% CI** | **p** |
| --- | --- | --- | --- |
| Marital Status |  |  |  |
| Living with a spouse (ref) | 1 | 1 |  |
| Separated/divorced/bereaved | 1.79 | 1.26, 2.55 | 0.001 |
| Single | 1.47 | 0.94, 2.30 | 0.084 |
| Age |  |  |  |
| 30-39(ref) | 1 | 1 |  |
| 40-49 | 1.59 | 1.17, 2.16 | 0.003 |
| 50-64 | 4.09 | 3.04, 5.49 | <0.001 |
| Drinking experience for the past year |  |  |  |
| No | 1.47 | 1.21, 1.77 | <0.001 |
| Yes (ref) | 1 | 1 |  |
| Smoking |  |  |  |
| Non-smoker (ref) | 1 | 1 |  |
| Former smoker | 0.97 | 0.77, 1.22 | 0.818 |
| Smoker | 1.36 | 1.06, 1.74 | 0.015 |
| BMI |  |  |  |
| Underweight (ref) | 1 | 1 |  |
| Normal weight | 1.10 | 0.59. 2.06 | 0.743 |
| Pre-obesity class | 1.22 | 0.63, 2.35 | 0.538 |
| Class 1 obesity | 1.69 | 0.86, 3.30 | 0.123 |
| Class 2 obesity or higher | 3.80 | 1.80, 8.01 | <0.001 |
| WHtR |  |  |  |
| <0.5 (ref) | 1 | 1 |  |
| 0.5≤ | 1.37 | 1.04, 1.81 | 0.023 |
| High cholesterol |  |  |  |
| No (ref) | 1 | 1 |  |
| Yes | 2.03 | 1.66, 2.49 | <0.001 |
| Hypertension |  |  |  |
| Normal (ref) | 1 | 1 |  |
| Pre-hypertension | 1.34 | 1.07, 1.67 | 0.008 |
| Hypertension | 1.31 | 1.03, 1.66 | 0.024 |
